# OmniSearch: a semantic search system based on the Ontology for MIcroRNA Target (OMIT) for microRNA-target gene interaction data

**DOI:** 10.1186/s13326-016-0064-2

**Published:** 2016-05-10

**Authors:** Jingshan Huang, Fernando Gutierrez, Harrison J. Strachan, Dejing Dou, Weili Huang, Barry Smith, Judith A. Blake, Karen Eilbeck, Darren A. Natale, Yu Lin, Bin Wu, Nisansa de Silva, Xiaowei Wang, Zixing Liu, Glen M. Borchert, Ming Tan, Alan Ruttenberg

**Affiliations:** School of Computing, University of South Alabama, Mobile, Alabama, 36688-0002 USA; Computer and Information Science Department, University of Oregon, Eugene, Oregon, 97403-1202 USA; Miracle Query, Inc., Eugene, Oregon, 97403-1202 USA; Department of Philosophy, University at Buffalo, Buffalo, New York, 14260-4150 USA; Genome Informatics, The Jackson Laboratory, Bar Harbor, Maine, 04609-1523 USA; Department of Biomedical Informatics, University of Utah, Salt Lake City, Utah, 84112-5775 USA; Department of Biochemistry and Molecular and Cellular Biology, Georgetown University Medical Center, Washington D.C., 20007-1485 USA; Center for Computational Science, University of Miami, Miami, Florida, 33146-2960 U.S.A; Department of Microbiology and Immunology, First Affiliated Hospital, Kunming Medical University, Kunming, Yunnan, 650032 China; Department of Radiation Oncology, Washington University School of Medicine, St. Louis, Missouri, 63110-0001 USA; Mitchell Cancer Institute, University of South Alabama, Mobile, Alabama, 36604-1405 USA; Department of Biology, University of South Alabama, Mobile, Alabama, 36688-0002 USA; School of Dental Medicine, University at Buffalo, Buffalo, New York, 14214-8006 USA

**Keywords:** microRNA, Non-coding RNA, Target gene, Biomedical ontology, Ontology development, Data annotation, Data integration, Semantic search, SPARQL query

## Abstract

As a special class of non-coding RNAs (ncRNAs), microRNAs (miRNAs) perform important roles in numerous biological and pathological processes. The realization of miRNA functions depends largely on how miRNAs regulate specific target genes. It is therefore critical to identify, analyze, and cross-reference miRNA-target interactions to better explore and delineate miRNA functions. Semantic technologies can help in this regard. We previously developed a miRNA domain-specific application ontology, Ontology for MIcroRNA Target (OMIT), whose goal was to serve as a foundation for semantic annotation, data integration, and semantic search in the miRNA field. In this paper we describe our continuing effort to develop the OMIT, and demonstrate its use within a semantic search system, OmniSearch, designed to facilitate knowledge capture of miRNA-target interaction data. Important changes in the current version OMIT are summarized as: (1) following a modularized ontology design (with 2559 terms imported from the NCRO ontology); (2) encoding all 1884 human miRNAs (vs. 300 in previous versions); and (3) setting up a GitHub project site along with an issue tracker for more effective community collaboration on the ontology development. The OMIT ontology is free and open to all users, accessible at: http://purl.obolibrary.org/obo/omit.owl. The OmniSearch system is also free and open to all users, accessible at: http://omnisearch.soc.southalabama.edu/index.php/Software.

## Introduction

microRNAs (miRNAs) are a type of non-coding RNA (ncRNA) with important biological, biomedical, and clinical impact. Prior research [1, 2] indicates that miRNAs perform significant roles in both biological and pathological processes, thus affecting the control and regulation of various human diseases. miRNAs realize critical functions via binding to their respective target genes. The ability to identify and analyze miRNA-target interactions in an effective manner is thus a key step in the understanding and delineation of miRNA functions.

The conventional method by which the users of data (e.g., biologists, bioinformaticians, and clinical investigators) determine miRNA functions involves:

- Searching for biologically validated miRNA targets, for example, by querying the PubMed database [3]; and
- Finding additional potential miRNA targets, for example, by initiating inquiries on various prediction databases or websites such as miRDB [4], TargetScan [5], and miRanda [6].

Unfortunately, both steps currently require significant manual effort because the relevant data sources are both syntactically and semantically heterogeneous — that is, the meaning of seemingly similar data from different sources may be quite different and thus open to misinterpretation. It is therefore challenging for users to identify and establish possible links among original data sources. As a result, conventional miRNA knowledge discovery and acquisition methodologies are time-consuming, labor-intensive, error-prone, and sensitive to limitations in the prior knowledge of different end users. These barriers are exacerbated by the need to obtain additional information for each and every miRNA target (whether validated or putative) using existing data sources and analysis tools, including but not limited to: the DAVID Bioinformatics Resources (DAVID) [7], NCBI Gene [8], the Medical Subject Headings (MeSH) Database [9], the HUGO Gene Nomenclature Committee (HGNC) Database [10], and NCBI Nucleotide [11].

Emerging semantic technologies can help in addressing the aforementioned challenges. The core of current semantic technologies include specifications such as the resource description framework (RDF), RDF Schema (RDFS), and Web Ontology Language (OWL), all of which are intended to provide a formal description of classes of entities of different types and of the relations between them in such a way as to enable automatic reasoning (inference). Semantic technologies can be applied to miRNA knowledge acquisition by transforming data obtained from heterogeneous miRNA-related databases into a common framework by utilizing a single format (such as RDF) and aligning the data through use of annotations from common, formally defined ontologies. By means of this transformation we can use the SPARQL Protocol (SPARQL) [11] to query the enhanced data automatically.

In previous research [12–17], we investigated the construction of an application ontology for the miRNA field, named Ontology for MIcroRNA Target (OMIT), the first ontology to formally encode miRNA domain knowledge. By providing a standardized metadata model to establish miRNA data connections among heterogeneous sources, the OMIT is able to fill two gaps: the lack of common data elements and the lack of data exchange standards for miRNA research, especially with regard to miRNA-target interactions.

We describe two major scientific contributions in this paper: (1) recent improvements to the OMIT ontology and (2) a semantic search system, which is built upon the ontology and enables the capture of miRNA-target interaction data in a way leading to more effective miRNA knowledge acquisition.

The remainder of this paper is organized as follows. “Related work” Section summarizes state-of-the-art research in biomedical ontologies and semantic search, respectively. “OMIT reconstruction” Section reports our efforts on reconstructing the OMIT ontology. “OmniSearch: an OMIT-based semantic search system” Section describes technical details of OmniSearch, an OMIT-based semantic search system. “Results and discussion” Section reports our experimental results. Finally, “Conclusions” Section summarizes the major points and presents ideas for future research.

## Related work

### Related work in biomedical ontologies

The use of ontologies to describe, define, and integrate biological entities has long been embraced by the biological, biomedical, and clinical research communities. Here we briefly describe some representative bio-ontologies included in both the Open Biological and Biomedical Ontologies (OBO) Library [18] and the National Center for Biomedical Ontology (NCBO) BioPortal [19] that are pertinent to the development of this project.

The Gene Ontology (GO) [20] is by far the most successful and widely used ontology for biological data description. It consists of three independent sub-ontologies: biological processes, molecular functions, and cellular components, which describe these aspects of gene products: both protein and RNA. The GO has been widely utilized to annotate gene products of model organisms. By the time of writing this paper, there were GO annotations for 36 organisms including Homo sapiens available for download.

The Sequence Ontology (SO) [21] is an ontology to capture genomic features and the relationships that obtain between them. This ontology contains the features necessary to annotate a genome with structural features such as gene models and also the terms necessary for the annotation of genomic variants. SO terms define the kinds of and parts of ncRNA features, and these terms are used to identify these features and their location in genomic sequence.

The PRotein Ontology (PRO) [22] is a comprehensive description of the forms of protein, including isoforms, modifications, and the relationships between them. Proteins are functional entities in many processes eventually impacted by the regulatory effect of ncRNAs (e.g., miRNA bindings). The PRO provides an ontological representation of proteins with a particular focus on human proteins and disease-related variants thereof.

The RNA Ontology (RNAO) [23] is a candidate OBO foundry reference ontology to catalogue the molecular entities composing primary, secondary, and tertiary components of RNA. The goal of this project is to enable integration and computation over diverse RNA datasets.

### Related work in semantic search

Semantic search is a research field that intends to improve the access to contents by considering the semantics behind the search process [24]. In other words, semantic search goes beyond conventional, keyword-based search by considering the contextual meaning of words, the intent of the user, and the nature of the search space. In general, semantic search requires the use of structured knowledge, such as ontologies, in the modeling and interpretation of queries. Ontologies can help improve the search by query expansion. One main idea in many semantic search systems (e.g., [25–29]) is, the original set of query keywords can be expanded by drawing on synonyms and other relationships (e.g., subclass and parthood) that are not part of the query. For example, in the work by Chauhan et al. [29], the original query was first expanded by considering synonyms, then terms with high semantic similarity were chosen from the ontology to be integrated to the search query, and the semantic similarity used for the query expansion was computed by the distance among concepts in the ontology, the position in the hierarchy, and the number of upper classes.

Another way to implement semantic search is to use ontologies to translate keyword-based search into formal semantic queries. For example, Tran et al. [24] used a set of models (mental, user, system, and query) to capture information, such as thought entities, language primitives, knowledge representation (KR) primitives, and query elements. These models were then combined with a set of assumptions to redefine original queries, filling the gap between terms with structural information from an ontology. That is, each term within the query was considered a property of another term.

## OMIT reconstruction

### Modularized ontology design

The OMIT ontology consists of the following modules:

- omit.owl — Defines all OMIT-specific terms and relations, for example, *prediction_from_miRDB* and *gene_context_score_in_TargetScan*.
- bfo.owl — Imports upper-level terms from the Basic Formal Ontology (BFO) [30], for example, *generically dependent continuant* and *material entity*.
- ro-imports.owl — Imports common relations (shared across different ontologies) from the Relation Ontology (RO) [31], for example, *has participant* and *regulates*.
- ncro.owl — Imports ncRNA-related terms and relations from the Non-coding RNA Ontology (NCRO) [32], for example, *miRNA_target_gene* and *miRNA_gene_family*.
- go-imports.owl — Imports gene product terms from the GO, for example, *RNA binding* and *regulation of biological process*.
- so-imports.owl — Imports sequence structural feature terms from the SO, for example, *biological_region* and *insertion_site*.
- obi-imports.owl — Imports life-science and clinical investigation terms from the Ontology for Biomedical Investigations (OBI) [33], for example, *cultured cell population* and *organism*.
- chebi-imports.owl — Imports molecular entity (especially small chemical compounds) terms from the Chemical Entities of Biological Interest Ontology (ChEBI) [34], for example, *ribonucleic acid* and *ribosomal RNA*.
- iao-imports.owl — Imports information entity terms from the Information Artifact Ontology (IAO) [35], for example, *information content entity*.
- clo-imports.owl — Imports cell line-relevant terms from the Cell Line Ontology (CLO) [36], for example, *cell line*.
- pr-imports.owl — Imports protein-related entity terms from the PRO, for example, *amino acid chain* and *protein*.
- uberon-imports.owl — Imports cross-species anatomy terms from the Uberon multi-species anatomy ontology (UBERON) [37], for example, *anatomical structure* and *organ*.
- doid-imports.owl — Imports disease terms from the Human Disease Ontology (DOID) [38], for example, *disease of cellular proliferation* and *cancer*.

Note that:

1. Orthogonality among different ontologies is one of the important practices proposed by the OBO Foundry Initiative, and has been widely accepted in the bio-ontology community. As a result, to achieve better orthogonality, it is a common practice to reuse contents defined in relevant, existing ontologies.
2. The OMIT ontology directly imported the NCRO ontology (a comprehensive ncRNA domain ontology), which in turn, directly imported other ontologies in the above list. Therefore, the OMIT ontology itself includes two OWL files: “omit.owl” and “ncro.owl.” All other OWL files, “go-imports.owl” and “so-imports.owl” for example, are shown as “*indirectly imported*” in Protégé.
3. Ontology concepts are referred to as “classes” in Protégé and “terms” in OBO Edit, respectively. Therefore, “classes” and “terms” are interchangeably used throughout the whole paper.

Table 1 lists a subset of important terms and relations imported into the OMIT.

**Table 1:** A subset of imported terms and relations

Imported term or relation	Source ontology		Original ID
RO:part of	Relation Ontology		BFO_0000050
RO:participates in	Relation Ontology		RO_0000056
RO:has participant	Relation Ontology		RO_0000057
BFO:entity	Basic Formal Ontology		BFO_0000001
BFO:continuant	Basic Formal Ontology		BFO_0000002
BFO:independent continuant	Basic Formal Ontology		BFO_0000004
BFO:occurrent	Basic Formal Ontology		BFO_0000003
BFO:material entity	Basic Formal Ontology		BFO_0000040
CHEBI:molecular entity	Chemical Entities of Biological Interest Ontology		CHEBI_23367
CHEBI:ribonucleic acid	Chemical Entities of Biological Interest Ontology		CHEBI_23367
CHEBI:ribosomal RNA	Chemical Entities of Biological Interest Ontology		CHEBI_18111
CHEBI:small nuclear RNA	Chemical Entities of Biological Interest Ontology		CHEBI_74035
CHEBI:transfer RNA	Chemical Entities of Biological Interest Ontology		CHEBI_17843
NCRO:human_miRNA	Non-coding RNA Ontology		NCRO_0000810
NCRO:hsa-miR-125b-1-3p	Non-coding RNA Ontology		NCRO_0003283
NCRO:hsa-miR-125b-2-3p	Non-coding RNA Ontology		NCRO_0003284
NCRO:hsa-miR-125b-5p	Non-coding RNA Ontology		NCRO_0003282
NCRO:miRNA_target_gene	Non-coding RNA Ontology		NCRO_0000025
NCRO:miRNA_and_target_gene_binding	Non-coding RNA Ontology		NCRO_0000003
NCRO:protein_miRNA_promoter_binding	Non-coding RNA Ontology		NCRO_0000011
IAO:information content entity	Information Artifact Ontology		IAO_0000030
IAO:measurement datum	Information Artifact Ontology		IAO_0000109

- The format for the left column (Imported Term or Relation) is PREFIX:human-readable label, for example, NCRO:miRNA_target_gene and RO:part of.
- The format for the right column (Original ID) is PREFIX_unique identifier, for example, NCRO_0000025 and BFO_0000001.

### Ontology core design

**Fig. 1:**
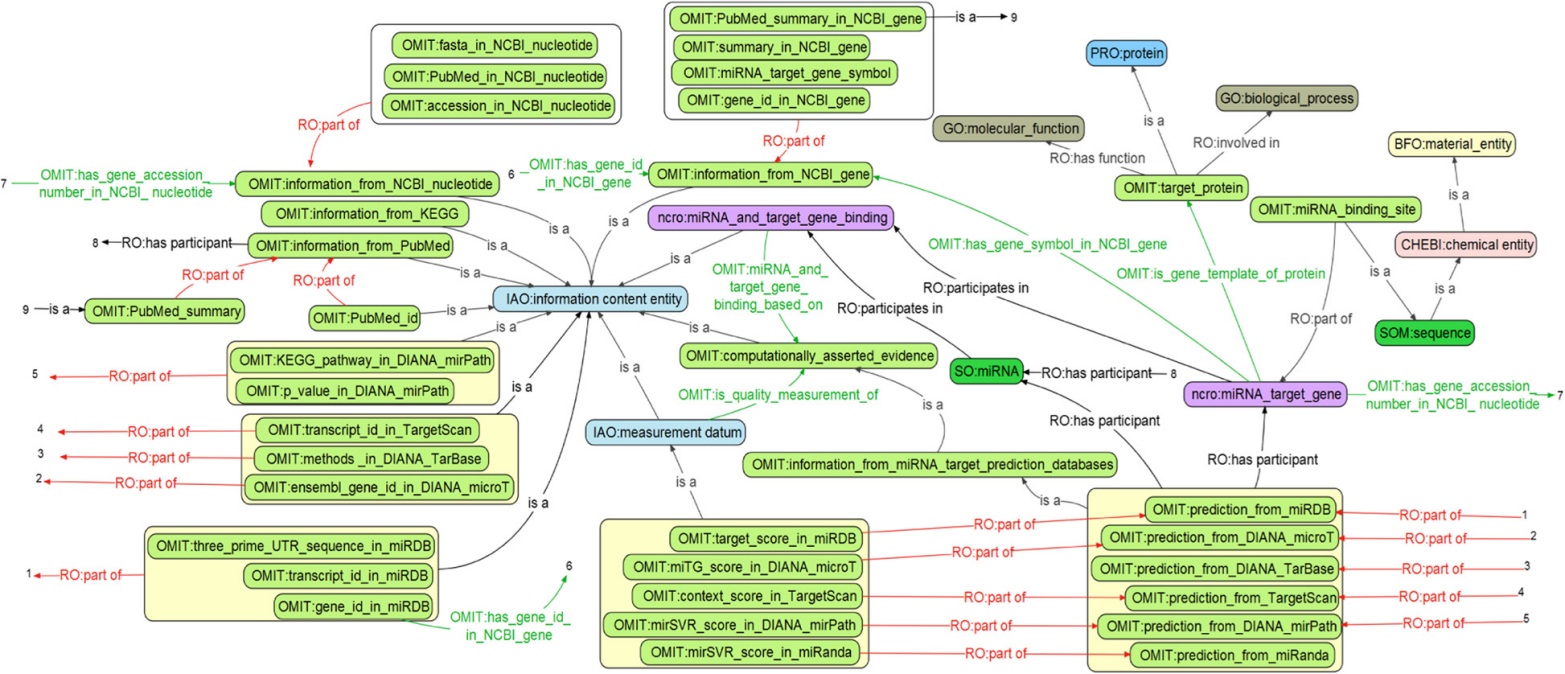
The design of core terms and relations in the OMIT ontology (both terms and relations are represented in the format of *PREFIX:label*)

The core design of the OMIT ontology is shown in Fig. 1. Compared with earlier versions, the current version contains many important new terms and relations, and some of which are listed in Tables 2 and 3, respectively.

**Table 2:** A subset of new OMIT terms

OMIT new term	Direct parent term	Human-readable explanation
computationally_asserted_evidence	IAO:information content entity	Evidence obtained from some
		computational methods.
information_from_miRNA_	OMIT:computationally_asserted_evidence	Records obtained from various
target_prediction_database		miRNA target prediction databases.
prediction_from_miRDB	OMIT:information_from_miRNA_	Records specifically obtained
	target_prediction_database	from the miRDB database.
prediction_from_TargetScan	OMIT:information_from_miRNA_	Records specifically obtained
	target_prediction_database	from the TargetScan database.
prediction_from_miRanda	OMIT:information_from_miRNA_	Records specifically obtained
	target_prediction_database	from the miRanda database.
target_score_in_miRDB	IAO:measurement datum	The score of some specific
		miRNA-target binding prediction
		from the miRDB database.
gene_context_score_in_TargetScan	IAO:measurement datum	The context score of some specific
		miRNA-target binding prediction
		from the TargetScan database.
mirSVR_score_in_miRanda	IAO:measurement datum	The mirSVR score of some specific
		miRNA-target binding prediction
		from the miRanda database.
information_from_NCBI_gene	IAO:information content entity	Records obtained from NCBI Gene
		according to gene IDs or gene symbols.
information_from_NCBI_nucleotide	IAO:information content entity	Records obtained from NCBI Nucleotide
		according to GenBank Accession numbers.
information_from_PubMed	IAO:information content entity	Records obtained from the PubMed
		database according to PMIDs.

**Table 3:** A subset of new OMIT relations

New relation	Domain	Range	Human-readable explanation
OMIT:miRNA_target_	NCRO:miRNA_and_	OMIT:computationally_	Specific miRNA-target binding
assumption_	target_gene_binding	asserted_evidence	prediction is based on some
based_on			computationally asserted evidence.
OMIT:is_quality_	IAO:measurement datum	OMIT:computationally_	A piece of measurement datum
measurement_of		asserted_evidence	(e.g., the target score in miRDB)
			is a quality measurement of
			computationally asserted evidence.
OMIT:is_gene_	NCRO:miRNA_target_gene	OMIT:target_protein	A miRNA target gene
template_of_protein			serves as a template
			of relevant protein.
RO:has participant	OMIT:prediction_from_miRDB	SO:miRNA	Each miRNA-target binding
			prediction record has one
			miRNA as a participant.
RO:has participant	OMIT:prediction_from_miRDB	NCRO:miRNA_target_gene	Each miRNA-target binding
			prediction record has one
			target as a participant.
RO:part of	OMIT:target_score_in_miRDB	OMIT:prediction_from_miRDB	Each miRNA-target binding
			prediction record from
			miRDB contains one score.
			Each record from NCBI
RO:part of	OMIT:PubMed_summary_	OMIT:information_from_NCBI_gene	Gene contains one or
		in_NCBI_gene	more PubMed summaries.

- Both terms and relations are represented in the format of *PREFIX:label* in Fig. 1.
- For the purpose of better readability, labels rather than identifiers are used in Tables 2 and 3.
- Relations in Table 3 were either defined in or imported into the OMIT, which can be easily distinguished from each other by different prefixes used in the first column.

## OmniSearch: an OMIT-based semantic search system

Based on the OMIT ontology, we developed a semantic search system: *OmniSearch*. First, the OmniSearch system will conduct semantic annotation on various sources that were originally heterogeneous in their semantics; following that, OMIT-annotated data will then be integrated into a unified and consistent data layer in RDF; and finally, complex semantic queries will be performed to provide meaningful results and clues to system end users (e.g., biologists, bioinformaticians, and clinical investigators).

### Data sources used

Data sources used in the OmniSearch system include three miRNA target prediction databases (miRDB, TargetScan, and miRanda), as well as PubMed, NCBI Gene, GO, RNA Central, DAVID, HGNC, and MeSH term databases. These sources contain both structured data (database instances) and unstructured data (free text), and are semantically heterogeneous among each other.

### Software architecture

The OmniSearch system consists of several software modules: semantic annotation, data integration, and semantic search.

Semantic data annotation is the process of tagging source files with predefined ontological metadata like names, entities, attributes, definitions, and descriptions. The annotation provides original data with extra metadata information formally defined in the OMIT ontology. The output of semantic data annotation is a collection of RDF triples (from both free text and database instances). These triples will be accumulated into a centralized RDF repository: OmniStore.

**Fig. 2:**
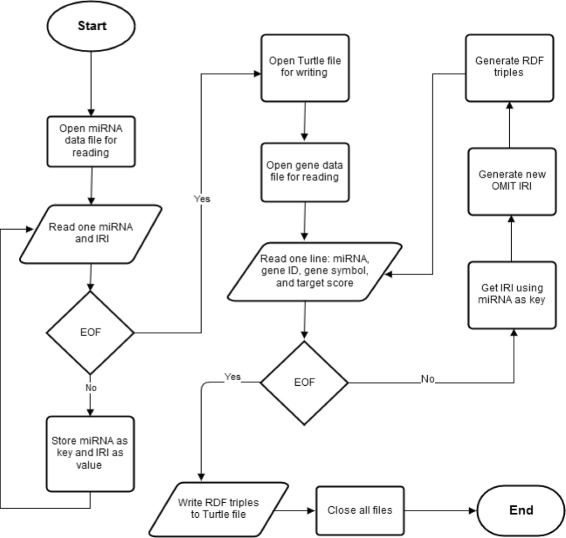
Semantic annotation and data integration flowchart in the OmniSearch system

We used Python scripts to conduct automated semantic annotation and data integration. As an example, Fig. 2 shows the flowchart of our programs to annotate miRDB data. We explain below the detailed steps. One miRDB file, the “miRNA data” file, contains two columns consisting of miRNA names and their associated internationalized resource identifiers (IRIs). Another miRDB file, the “gene data” file, contains four columns consisting of miRNA names, gene IDs, gene symbols, and target scores.

- Step One: As each miRNA name and its associated IRI were read in from the miRNA data file, they were placed into a dictionary where the miRNA name is the key and the IRI is the value.
- Step Two: All lines were read in from the gene data file, and each line was converted into a total of four RDF triples. (1) The first triple was generated to represent a newly created instance of the *prediction_from_miRDB* class, namely, *instance_i*, and a new OMIT IRI was assigned to *instance_i*. (2) Next, the miRNA name read from the same line was used to retrieve its corresponding IRI from the dictionary (generated in Step One). The second triple then connected this retrieved IRI with *instance_i*. (3) Two more triples were generated to connect *instance_i* with the corresponding gene ID and target score read in from the same line, respectively.
- Step Three: Finally, all generated RDF triples were written into a Turtle file.

Note that:

1. “Semantic annotation and data integration” Section exhibits some example triples resulted from the above-mentioned annotation process.
2. Mappings between database schemas and ontological entities were defined in the OMIT ontology and can be reused or modified in the future, when needed.
3. Due to our automated annotation and integration techniques, only minimum effort will be required to integrate a new resource in the future.

**Fig. 3:**
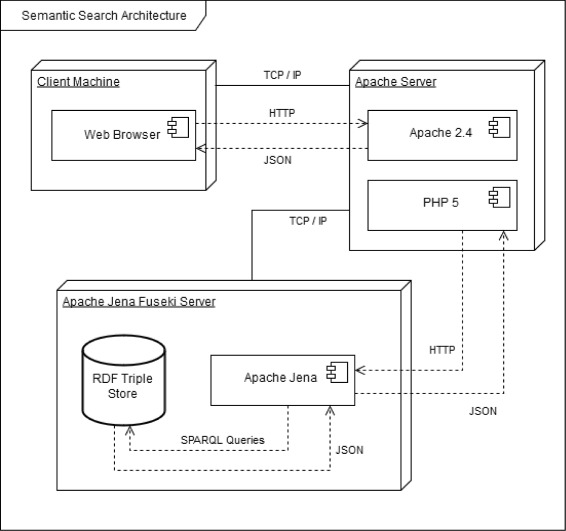
Semantic search architecture in the OmniSearch system

Because all semantic tags are to be generated from the global metadata model defined in the OMIT ontology, the RDF triple repository will provide a unified view over original data sources at semantic level. Consequently, complex semantic queries will be enabled. To implement semantic search, we made use of Apache HTTP server [39], PHP: Hypertext Preprocessor (PHP) server [40], and Apache Jena Fuseki server [41]. The overall software architecture is demonstrated in Fig. 3, with the following working protocol:

- Query parameters are sent from the client’s browser to the Apache server through Ajax requests.
- SPARQL queries are dynamically generated by the Apache server using these query parameters, which are then sent to the Apache Jena Fuseki server.
- JSON objects, containing the requested information, are retrieved from the RDF triple store (installed on the Apache Jena Fuseki server) after running the dynamically generated SPARQL queries.
- These JSON objects are returned to the Apache server, which are used to generate either (1) a list of miRNAs and/or MeSH terms or (2) the HTML Markup for the search result table.
- Finally, the Apache server sends the obtained data, or an error message if the search fails, back to the client’s browser as a JSON object.

### User interface design

**Fig. 4:**
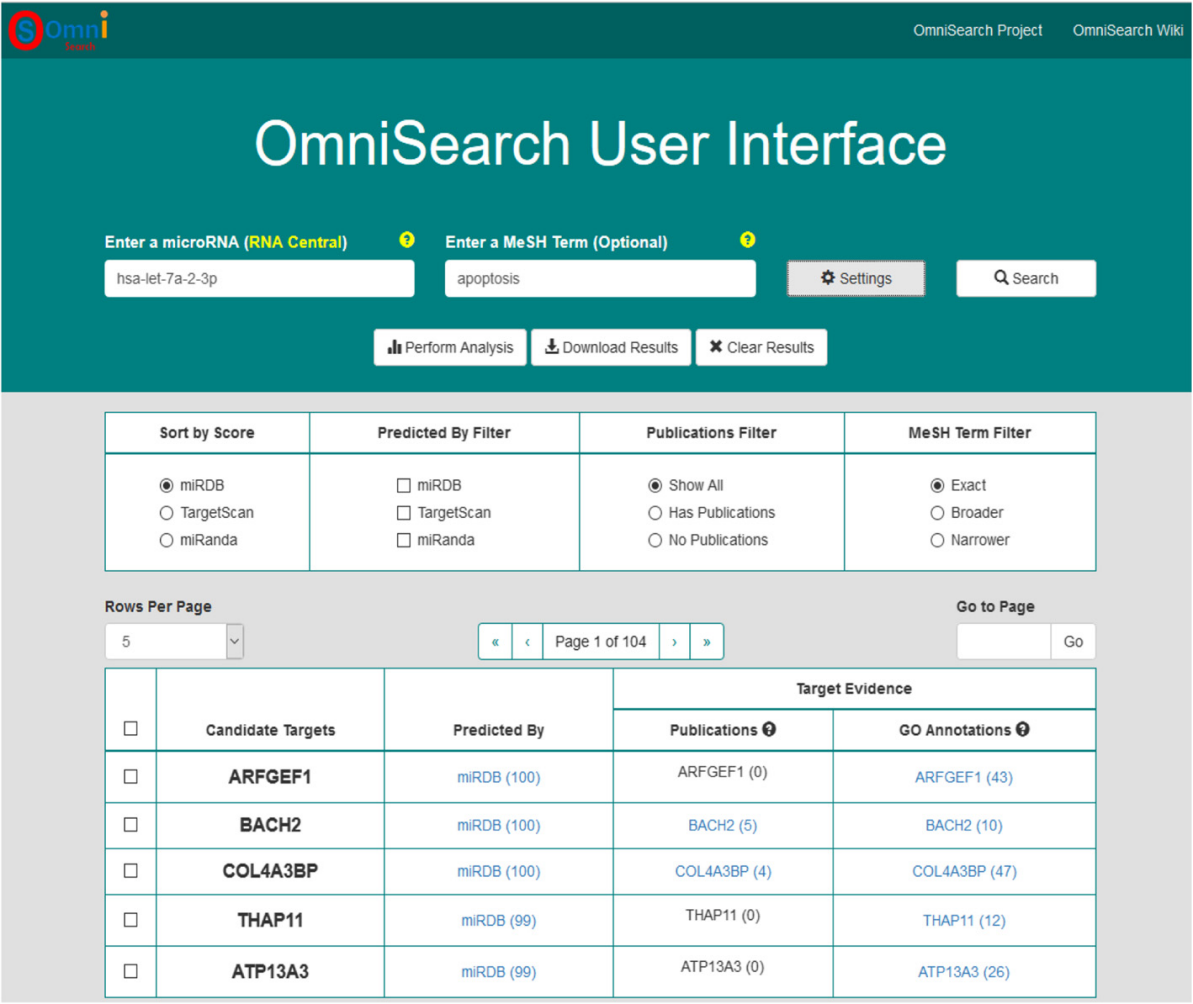
GUI design in the OmniSearch system

The OmniSearch is a Web-based search system that is free and open to all users, accessible at: http://omnisearch.soc.southalabama.edu/index.php/Software. As shown in Fig. 4, the main components of the graphic user interface (GUI) are: two search criteria boxes, a search result table, a pagination control, a set of result viewing filters, a result download tool, and DAVID analysis functionality. More discussion on our friendly user interface design can be found in “Search results and discussion” Section.

## Results and discussion

### The significantly refactored OMIT ontology

The updated version of the OMIT ontology contains a total of 3169 terms and 46 relations (besides a total of 5515 *is_a* relations). Note that out of 46 relations mentioned here, there are 5 data properties, and the rest are object properties. Also note that these terms and relations include both OMIT-specific ones and those imported ones^1^.

Compared with the previous versions [12–17], important changes in the current version OMIT ontology are summarized as follows.

- As discussed earlier in “Modularized ontology design” Section, we have followed a ***modularized ontology design*** in this new version, which will significantly further facilitate the ontology maintenance and update. In particular, a total of 2559 terms in the updated OMIT have been imported from the NCRO ontology [32]. Because the NCRO is a comprehensive domain ontology in the ncRNA field, following the NCRO hierarchy will enhance the interoperability between the OMIT and future ontologies to be developed in other ncRNA sub-domains.
- In the previous versions of OMIT, around 300 human miRNAs were included. In the current version, ***all 1884 miRNAs appearing in humans*** have been encoded, along with the information about the gene family group of each and every miRNA. According to miRBase [42], there are a total of 320 different gene family groups. This information can be highly valuable because the fact that two or more miRNAs of interest indeed belong to the same gene family group can provide biologists, bioinformaticians, and clinical investigators with critical clues in constructing new hypothesis.
- In our previous investigations, we established a dedicated project website [43], as well as entries in both the OBO Library [44] and the NCBO BioPortal [45]. To further disseminate the ontology, and, to gather feedback from community in a more effective manner, we have recently created ***a GitHub project site*** (https://github.com/OmniSearch/omit) for this new version OMIT ontology. We have also established ***a tracker*** [46] for an enhanced mechanism in handling the discussion among groups to further improve the ontology. New concepts, definitions, and their locations in the OMIT can now be proposed, debated, and approved (or rejected) by an open group of individuals through this tracker.

### Semantic annotation and data integration

#### Experimental setup

The OmniStore RDF repository is housed on a server with the following configuration: Intel(R) Core(TM) i7-3632 QM CPU @ 2.80 GHz 2.80 GHz; 32.00 GB memory; and Windows Server 8 Operating System.

#### Semantic annotation and data integration results

OmniStore contains a total of 6,136,514 RDF triples, and the file size of OmniStore is 369 MB. All triples are represented in RDF 1.1 Turtle: Terse RDF Triple Language format [47], for example:



The semantics of the above six example triples is: IRF4 (OMIT_0015037) is a subclass of the *miRNA_target_gene* class (NCRO_0000025); one miRDB database record (OMIT_0995324), which is an instance of the *prediction_from_miRDB* class (OMIT_0000020), indicates that IRF4 is a predicted target of the miRNA *hsa-miR-125b-5p* (OMIT_0050688); and the prediction score (OMIT_0000108) is 100.

### Semantic search

We use one example in this section to demonstrate in detail how the OmniSearch system assists in end users’ knowledge acquisition.

#### Experimental setup

Semantic search was conducted on a personal computer (PC) with the following configuration: Intel(R) Core(TM) i7-3632 QM CPU @ 2.50 GHz 2.50 GHz; 16.00 GB memory; and Windows 10 64-bit Operating System.

#### SPARQL query statements

The SPARQL statements to generate the miRNA and MeSH term lists in the two search boxes are as follows, where the PHP variable *$type* is used to determine whether the client is requesting a miRNA or MeSH term, and the PHP variable *$input* contains either a partial or exact miRNA or MeSH term. Note that each line of the query statement has a detailed explanation right above it (the line starting with a pound sign “#”).



Suppose that the question of interest is: “What is the role of hsa-miR-125b-5p in cancer drug resistance?” The SPARQL statements are as follows. Similarly, all query statements have a detailed explanation.





#### Search results and discussion

**Fig. 5:**
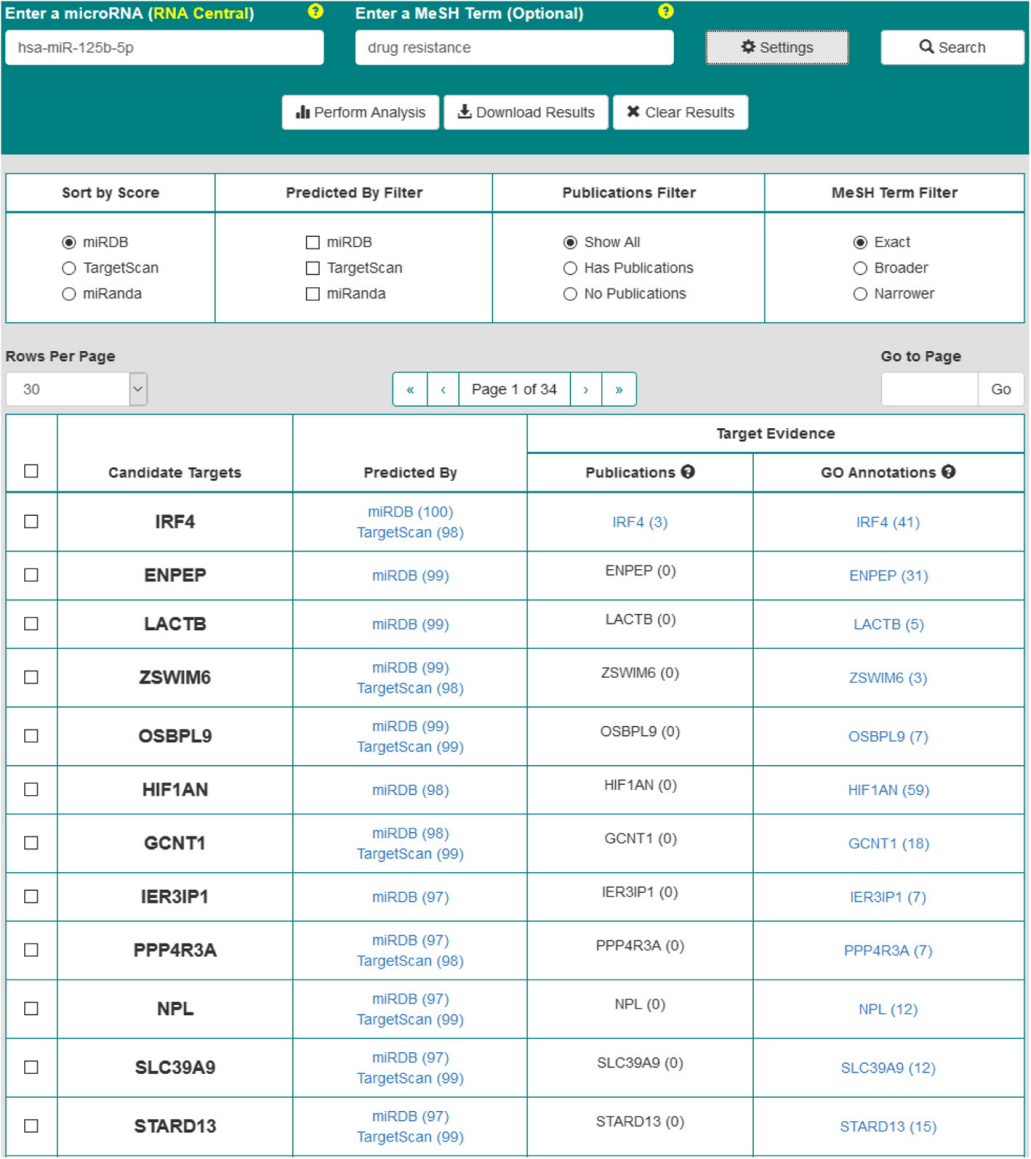
Search results for the question of “What is the role of hsa-miR-125b-5p in cancer drug resistance?”

Corresponding to the aforementioned question of interest, Fig. 5 demonstrates the search results from a query on *hsa-miR-125b-5p* along with a MeSH-term filter “drug resistance”.

- The “Candidate Targets” column contains all targets predicted by at least one target prediction database. The user can choose a prediction database and sort all targets by the scores, in descending order, from the selected database.
- The “Predicted By” column shows that each target is predicted by which database(s), along with the Web link(s) to these database(s).
- The “Publications” column links to all PubMed publications that are relevant to the search and filtering criteria. In this example, the criteria for any line are: the predicted target on that line, the miRNA *hsa-miR-125b-5p*, and the MeSH-term filter “drug resistance.”
- The “GO Annotations” column connects to GO annotation results of each predicted target and the miRNA *hsa-miR-125b-5p*, respectively.
- Pathway analysis through DAVID can be performed on selected targets, either using the checkboxes to the left of the table or clicking the “Select All Targets” checkbox. Additionally, the user can select the desired tool to perform such analysis, “Gene Functional Classification,” “Functional Annotation Clustering,” “Functional Annotation Summary,” and so forth.
- The whole result table can be downloaded in two different formats (tab-delimited text or CSV format); the user is also able to download only the predicted targets (selected ones or all).

We examined the search results demonstrated in this example, and our observations are summarized below.

1. Effective querying and accurate search results.
  Potential targets from all three miRNA target prediction databases (miRDB, TargetScan, and miRanda) were correctly retrieved. There were 476 and 924 targets from miRDB and TargetScan, respectively; and there were 323 common targets. Consequently, a total of 1077 distinct targets were retrieved in the table when the “Predicted by Any Database” filter was chosen. Note that the miRanda database did not contain prediction results for the miRNA *hsa-miR-125b-5p*; therefore, no results appeared in the table when the display filter was set to “Predicted by All Databases.” In fact, this observation further verified the effectiveness of the OmniSearch system.
  Relevant papers according to the search criteria were successfully retrieved. For example, two publications (PMID: 2497002 and 22808086) were retrieved for the predicted target LIN28A, supporting the conclusion that “Lin28A contributes to cancer drug resistance;” and three publications (PMID: 21823019, 24643683, and 19463775) were retrieved for the predicted target BAK1, supporting another conclusion that “BAK1 has an important role in cancer drug response and drug resistance.”
  RNA Central annotations and GO annotations were correctly obtained. In this example query, a total of five sequences regarding the miRNA *hsa-miR-125b-5p* were retrieved from RNA Central annotations, and GO annotations for all predicted targets were retrieved as well. For example, a total of 117 GO annotations (GO_REF:0000038, GO_REF:0000033, and so forth) were retrieved regarding a potential target, BAK1.
  Based on the above knowledge returned in the OmniSearch GUI, regarding the example question of “What is the role of hsa-miR-125b-5p in cancer drug resistance?” end users obtained the following answer: ***It is reasonable to speculate that expression of the miRNA hsa-miR-125b-5p contributes to cancer drug resistance, possibly through its suppression of expression for target genes BAK1 and/or LIN28A***.
  *Discussion:*
  (1) miRDB, TargetScan, and miRanda databases have quite different meanings among each other in terms of their database entities. Due to the underlying OMIT and the formally defined semantics in the ontology, the OmniSearch system was able to effectively integrate the prediction results from all three databases. Note that conventional, database-oriented techniques can also implement such integration; however, inflexible, ad-hoc hard-coding will be required.
  (2) To retrieve a correct set of relevant papers requires accessing numerous heterogeneous data sources such as NCBI Gene, PubMed, HGNC, and MeSH. Without the common data elements defined in the OMIT and the thereafter semantic technologies including semantic annotation and data integration, it would have been extremely challenging to effectively integrate data from these sources, which is the case in database-oriented search and querying.
  (3) As discussed earlier in “OMIT reconstruction” Section, the OMIT is closely connected with the GO by importing a set of GO terms. Compared with data integration based on traditional, relational databases, our approach has further facilitated integrating data about GO annotations.
2. More efficient querying process.
  One-stop visit rather than accessing different data sources separately, resulting in about 60 % of time saved for end users.
  DAVID analysis was performed in a more efficient manner due to the target gene list automatically generated by the system. resulting in about 50 % of time saved for end users.
  It was easier to compare different prediction results among miRDB, TargetScan, and miRanda databases, resulting in about 60 % of time saved for end users.
  The above percentages of saved time were calculated as follows: We asked the aforementioned domain experts to perform a given set of queries using their conventional methods; next, they performed the same set of queries through the OmniSearch GUI; and finally, the saved time for all domain experts were averaged. Greater details on the system time and saved time for end users are contained in Table 4.

**Table 4:** The system time and saved time for end users

Query	First search	Second search	System time	User time	Percentage of saved	Percentage of saved	Percentage of saved
	criterion	criterion	(seconds)	(seconds)	time for end users	time on DAVID analysis	time on result comparison
1	hsa-miR-1231	cell movement	2.51	10	62 %	55 %	61 %
2	hsa-miR-1288-5p	cell proliferation	2.89	9	61 %	51 %	62 %
3	hsa-miR-143-3p	mitosis	5.54	10	61 %	52 %	60 %
4	hsa-miR-192-5p	leukemic infiltration	2.24	8	53 %	53 %	59 %
5	hsa-miR-216a-5p	drug resistance,	4.09	11	65 %	55 %	62 %
		multiple					
6	hsa-miR-29c-3p	recurrence	8.99	11	68 %	53 %	63 %
7	hsa-miR-3155a	dna cleavage	1.21	6	53 %	47 %	55 %
8	hsa-miR-320b	drug resistance	17.59	18	73 %	51 %	66 %
9	hsa-miR-3622a-5p	entosis	0.30	6	51 %	43 %	57 %
10	hsa-miR-371b-5p	mitochondrial	3.89	12	66 %	59 %	64 %
		dynamics					
11	hsa-miR-3934-5p	dna methylation	0.93	8	61 %	45 %	59 %
12	hsa-miR-4263	mutagenesis	1.65	6	52 %	46 %	56 %
13	hsa-miR-4431	mitochondrial	0.17	6	53 %	47 %	55 %
		degradation					
14	hsa-miR-4505	cell transformation,	4.25	10	63 %	55 %	61 %
		neoplastic					
15	hsa-miR-4648	cell polarity	0.71	6	52 %	45 %	57 %
16	hsa-miR-4700-3p	neoplasm regression,	1.56	7	53 %	51 %	59 %
		spontaneous					
17	hsa-miR-4756-5p	endocytosis	3.76	10	67 %	53 %	62 %
18	hsa-miR-4802-3p	drug resistance,	1.67	7	55 %	47 %	59 %
		microbial					
19	hsa-miR-501-3p	insulin resistance	1.78	8	57 %	43 %	61 %
20	hsa-miR-520a-3p	ubiquitination	13.31	17	75 %	55 %	65 %
Average	—————	—————	3.95	9.30	60.05 %	50.30 %	60.15 %
  Applying the MeSH-term filter resulted in a much smaller number of relevant publications returned. For example, 50 vs. 16 for the target ABCC5, 13 vs. 2 for the target DPH2, and 31 vs. 3 for the target FOXQ1. More examples are demonstrated in Table 5.

**Table 5:** Reduced number of publications after applying the MeSH-term filter “drug resistance”

Target gene	Original number	Number of papers	Percentage
symbol	of papers	after MeSH filtering	reduced
ABCC5	50	16	68 %
DPH2	13	2	85 %
FOXQ1	31	3	90 %
CIAPIN1	43	4	91 %
SLC38A9	12	1	92 %
MCL1	452	31	93 %
MKNK2	30	2	93 %
BAG4	32	2	94 %
ARID3B	18	1	94 %
HSPB2	79	4	95 %
THEMIS2	20	1	95 %
BAK1	266	11	96 %
SULT4A1	27	1	96 %
FUT4	57	2	96 %
GPC6	29	1	97 %
DDX54	29	1	97 %
MBD1	58	2	97 %
PRDM1	118	4	97 %
DTNB	30	1	97 %
LIN28A	91	3	97 %
SIRT7	33	1	97 %
ZBTB7A	67	2	97 %
NCOR2	240	7	97 %
TTPA	35	1	97 %
MAP3K10	35	1	97 %
SGPL1	36	1	97 %
MYO18A	36	1	97 %
EIF4EBP1	217	6	97 %
LIMK1	109	3	97 %
TP53INP1	37	1	97 %
CYTH1	39	1	97 %
SLC7A1	41	1	98 %
  *Discussion:*
  (1) The reduced time spent by users was due to ***both*** data integration ***and*** the more accurate semantics defined in the ontology.
  (2) In an non-ontology software system, to filtering on MeSH terms almost unavoidably results in hard-coding some ad-hoc searching rules in source code. On the contrary, semantics-oriented systems, such as OmniSearch, can well handle this issue in a more efficient manner. By decoupling domain knowledge from source code, ontologies and software applications can be developed independently, leading to more flexible software development.
  (3) Based on the *is_a* relation, the OmniSearch system can perform logic reasoning over the ontology concept hierarchy (that is, both broader and narrower terms of the ontology term of interest), thus greatly improving the flexibility of search and query capability. For example, after a MeSH term is chosen by users, they are able to search the exact MeSH term, or its broader terms (i.e., ancestor terms) and narrower terms (i.e., offspring terms) defined in the ontology. Such results would not have been obtained without semantic technologies because systems based on relational databases are not able to perform any logical reasoning. Of course, users can still manually perform numerous queries and then obtain similar results as obtained from our system. However, such manual querying is significantly more time-consuming and labor-intensive, and more importantly, error-prone.
  (4) Cross-referencing among miRDB, TargetScan, and miRanda prediction results was made much easier because relevant database entities have already been formally defined in the OMIT. In other words, unambiguous semantics was accurately encoded with common data elements provided by the ontology, resulting in successful data sharing and exchanging among heterogeneous data sources.
  (5) We asked the aforementioned domain experts to verify the accuracy of MeSH-term filtering. Because all returned publications contained the corresponding MeSH term, the *Precision* measure was evaluated as 100 %. As for the *Recall* measure, it took a much longer time to evaluate because we needed to identify all publications that were incorrectly filtered out by the system. For example, there were three (one, resp.) publications relevant to CSNK2A1 (DVL3, resp.) that should not have been filtered out. More such examples are demonstrated in Table 6. Overall, an average *Recall* of 73 % was achieved, meaning that while a user is able to obtain desired knowledge in a much more efficient manner (by reading significantly less publications, as shown in Table 5), the potential information lost is rather low.

**Table 6:** An example set of publications correctly/incorrectly filtered by “drug resistance”

Gene symbol	Total number of publications without applying the “drug resistance” filter	Total number of publications that contain the MeSH term “drug resistance”	Total number of incorrectly filtered publications
IRF4	130	3	0
ARID3B	18	1	0
SGPL1	36	1	0
ESRRA	131	3	0
PAFAH1B1	129	1	0
ETS1	287	5	0
TTPA	35	1	0
DVL3	60	1	1
THEMIS2	20	1	0
VTCN1	66	1	0
WDR5	128	1	0
ETV6	198	4	0
TAZ	74	1	0
IL6R	300	1	0
DPH2	13	2	0
BTG2	84	1	0
CYP24A1	146	2	0
LIN28A	91	3	0
TRPS1	69	1	0
CSNK2A1	619	5	3
TP53INP1	37	1	0
GPC6	29	1	0
DICER1	291	3	0
3. Friendly user interface.
  For both search boxes, a list of partially matching terms were presented in a drop-down box as users typed in the box. Users were also allowed to not to type in anything, in which case all terms will be presented.
  The “Rows Per Page” drop-down and pagination control helped users to easily navigate among all predicted targets.
  A set of display filters were designed to allow users to conveniently and freely customize their preferred way to view retrieved results from various facets. For example, results can be sorted by the prediction score from any selected prediction database; users can choose to view only results that have publication evidence, or does not have such evidence, or both; and so forth.
  Flexible download options were provided, and all downloaded documents had self-explanatory, meaningful file names that contain the search date, “Query_Results_for_hsa-miR-125b-5p-2015-12-05.csv” and “Target_List_for_hsa-miR-125b-5p-2015-12-05.txt” for example.

## Conclusions

As a special class of ncRNAs, miRNAs have been demonstrated to play important roles in various biological and pathological processes. Because miRNAs realize their functions by regulating respective targets, it is critical to identify and analyze miRNA-target interaction data to better explore and delineate miRNA functions. Semantic technologies and domain ontologies have been utilized to overcome limitations of conventional miRNA knowledge acquisition methods. To this end, we followed the research direction identified in our previous investigations regarding the establishment of common data elements and data exchange standards in the miRNA research. Specifically, our major scientific contributions in this paper are:

- We have significantly improved the OMIT ontology by: (1) following a modularized ontology design; (2) encoding all 1884 human miRNAs; and (3) setting up a GitHub project site along with an issue tracker for more effective community collaboration on the ontology development. The up-to-date ontology file is accessible at: http://purl.obolibrary.org/obo/omit.owl.
- Based upon the OMIT, we built the OmniSearch semantic search system, accessible at: http://omnisearch.soc.southalabama.edu/index.php/Software. Our experimental results demonstrated promising performance of OmniSearch. Consequently, more effective, more efficient miRNA-related knowledge capture has been achieved.

Finally, some research directions are envisioned as follows for our future work.

(1) To investigate a new set of filters to perform a wider scope of ontology reasoning. For example, potential filters can be developed according to different miRNA categories such as: oncogenic or tumor-suppressive miRNAs; individual tissues and/or cell lines in which miRNAs are expressed; and the gene family group to which miRNAs belong.

(2) To verify the consistency of contents retrieved from different data resources is another important future research topic. It is not trivial to resolve conflicting facts among different sources.

(3) It would be terrific for users to have more flexible options in further exploiting the semantics of the domain. Note that to construct more flexible queries will involve natural language processing (NLP) techniques, which are beyond the scope of this paper. Nevertheless, such an interesting topic can be considered in the future.

## Endnote

^1^ There are 103 and 18 OMIT-specific terms and relations, respectively.
